# Differential association of visceral adipose tissue with coronary plaque characteristics in patients with and without diabetes mellitus

**DOI:** 10.1186/1475-2840-13-61

**Published:** 2014-03-14

**Authors:** Kazuhiro Osawa, Toru Miyoshi, Yasushi Koyama, Shuhei Sato, Noriaki Akagi, Yusuke Morimitsu, Motoki Kubo, Hiroki Sugiyama, Kazufumi Nakamura, Hiroshi Morita, Susumu Kanazawa, Hiroshi Ito

**Affiliations:** 1 Department of Cardiovascular Medicine, Dentistry and Pharmaceutical Sciences, Okayama University Graduate School of Medicine, Okayama, Japan; 2 Department of Cardiovascular Therapeutics, Dentistry and Pharmaceutical Sciences, Okayama University Graduate School of Medicine, 2-5-1 Shikata-cho, Okayama 700-8558, Japan; 3 Cardiovascular Center, Sakurabashi Watanabe Hospital, Osaka, Japan; 4 Department of Radiology, Dentistry and Pharmaceutical Sciences, Okayama University Graduate School of Medicine, Okayama, Japan

**Keywords:** Visceral adipose tissue, Coronary artery disease, Diabetes mellitus

## Abstract

**Background:**

Excess visceral adipose tissue (VAT) is closely associated with the presence of coronary artery plaques that are vulnerable to rupture. Patients with diabetes mellitus (DM) have more VAT than patients without DM, but the extent to which VAT contributes to the characteristics of coronary plaques before and after the development of DM is not fully understood.

**Methods:**

We retrospectively evaluated 456 patients (60% male, age 64 ± 16 years) who were suspected to have cardiovascular disease and underwent 64-slice computed tomography angiography (CTA). Seventy-one (16%) patients had vulnerable plaques (CT density < 50 Hounsfield Units, positive remodeling index > 1.05, and adjacent spotty areas of calcification).

**Results:**

Patients were divided into tertiles according to the VAT area. There were stepwise increases in noncalcified and vulnerable plaques with increasing tertiles of VAT area in patients without DM, but not in patients with DM. Multivariate analysis showed that a larger VAT area was significantly associated with a higher risk of vulnerable plaque in patients without DM (odds ratio 3.17, 95% confidence interval 1.08–9.31, p = 0.04), but not in patients with DM.

**Conclusions:**

The VAT area is associated with the characteristics of coronary plaques on CTA in patients without DM, but not in patients with DM. VAT may be a significant cardiometabolic risk factor that is associated with plaque vulnerability before the development of DM. CTA findings may help to improve risk stratification in such patients.

## Background

Obesity is one of the most common causes of cardiovascular morbidity and mortality [1]. The body mass index (BMI) has been used to evaluate the impact of obesity on cardiovascular disease, but recent studies have shown that the amount of visceral adipose tissue (VAT) evaluated by the waist-to-hip ratio or computed tomography (CT) findings is more closely associated with cardiovascular events than BMI [2,3]. VAT is an endocrine organ that produces large amounts of adipokines. A large amount of VAT leads to adipose tissue dysfunction followed by impaired insulin sensitivity, secretion of pro-inflammatory adipokines, and ultimately type 2 diabetes mellitus (DM), left ventricular dysfunction, and coronary artery disease (CAD) [4-8].

Recent advances in cardiac CT angiography (CTA) technology have enabled noninvasive detection of noncalcified plaques (NCPs) and evaluation of the characteristics of vulnerable NCPs in terms of CT density, remodeling, and adjacent spotty areas of calcification [9,10]. Vulnerable plaques have an increased risk of rupture resulting in adverse coronary events [9,11]. The amount of VAT is closely associated with the characteristics of vulnerable coronary artery plaques [12-14].

Patients with DM have more VAT than those without DM [15,16], but the extent to which VAT contributes to the NCP burden and to the vulnerability of NCPs before and after the development of DM is not fully understood. The objective of this study was to evaluate the relationships between the characteristics of coronary plaques identified by CTA and the amount of VAT in patients with and without DM.

## Methods

### Study population

The study population included 502 consecutive patients who underwent 64-slice CT because of suspected stable coronary artery disease (effort angina pectoris or vasospastic angina pectoris) at Okayama University Hospital between August 2011 and December 2012. Patients with a history of coronary artery stenting (n = 28) or coronary artery bypass graft surgery (n = 6) were excluded. Patients in whom some coronary artery segments could not be evaluated because of motion artifacts or inadequate contrast filling (n = 16), or who had missing abdominal scans or missing information regarding one or more traditional coronary risk factors (n = 2), were also excluded. Some patients had more than one reason for exclusion, and 456 patients were included in this study. The study protocol was approved by the institutional ethics committee on human research of Okayama University. Written informed consent was obtained from all patients before inclusion in the study. The investigation conformed to the principles outlined in the Declaration of Helsinki.

### Multi-detector CT imaging protocol

Subjects underwent radiographic assessment of the chest and abdomen in the supine position during one procedure. CT imaging was performed using a Somatom Definition Flash scanner (Siemens Medical Solutions, Erlangen, Germany) [17]. The parameters were as follows: detector collimation 64 × 0.6 mm (equivalent to slice acquisition of 128 × 0.6 mm using the flying focal spot technique), table pitch adjusted to heart rate (0.17–0.38), rotation time 275 ms, tube current time product 360 mAs, and tube voltage 120 kVp. A test bolus of 10 mL of contrast medium followed by 20 mL of saline was injected at the level of the ascending aorta, and low-dose CT images were obtained every 1 s. The delay before the formal scan was calculated as the time to peak enhancement in the ascending aorta plus 3 s, to ensure enhancement of the distal segments of the coronary arteries. For the final scan, contrast agent (Omnipaque 350; Daiichi Sankyo, Tokyo, Japan) was injected over 10 s, followed by a second bolus of 80% of the amount of contrast medium diluted 50% and then a bolus of saline. All injections were administered at the same rate, calculated as the body weight × 0.07 mL/s. All patients arrived at the hospital 1 h before the scheduled CT time, and those with a persistent heart rate of ≥60 beats/min received oral metoprolol (20–40 mg). If the heart rate did not decrease to <60 beats/min before the scheduled CT time, patients received additional pre-medication such as oral metoprolol (20 mg), intravenous propranolol (2 mg), intravenous verapamil (5 mg), and/or intravenous landiolol hydrochloride (0.125 mg/kg) until the heart rate was <60 beats/min.

### CTA analysis

Coronary artery stenosis and plaques were evaluated on axial and curved multiplanar reformatted images using commercially available cardiac reconstruction software (Virtual Place, Raijin; AZE Inc., Tokyo, Japan). One experienced and trained senior cardiologist and two senior CT technicians performed the analyses, and evaluations were performed on a per-segment basis. Sixteen segments were identified based on the established American Heart Association segment model, and the presence and characteristics of coronary artery plaques on CTA were evaluated. The minimum CT density was measured in at least five regions of interest (each 1 mm^2^). Coronary plaques were defined as structures of > 1 mm^2^ within the coronary arteries that differed in density from the contrast-enhanced vessel lumen. Plaques were categorized as calcified plaques (> 130 Hounsfield Units [HU]), NCPs (< 130 HU), or low-density plaques (< 50 HU). Coronary artery remodeling was assessed by calculation of the difference in vessel diameter at the plaque site compared with a reference site in a normal-appearing segment proximal to the lesion, with positive remodeling defined as an index of > 1.05, as previously described [18]. An area of spotty calcification was defined as follows: length (in the longitudinal direction of the vessel) of the calcification < 3/2 of the vessel diameter, and width (perpendicular to the longitudinal direction of the vessel) of the calcification < 2/3 of the vessel diameter. Vulnerable plaque was defined as plaque with all of the following characteristics: positive remodeling, low density, and an adjacent area of spotty calcification. Significant coronary artery stenosis was defined as luminal obstruction of > 50% of the diameter of the vessel. The inter-observer coefficient of variation of 20 randomly selected samples was < 5%.

### Abdominal tissue measurements

Abdominal CT at the level of the umbilicus was performed at the same time as cardiac CT. The VAT area, subcutaneous adipose tissue (SAT) area, and waist circumference (WC) were assessed using Virtual Place software. WC was calculated automatically at the level of the umbilicus. Fat volume was measured using the semi-automatic segmentation technique [19]. The attenuation range for fat tissue was defined as the interval within 2 standard deviations of the mean in each individual. The muscular abdominal wall was manually traced to separate VAT from SAT.

### Assessment of other risk factors

DM was defined as self-reported history of DM, hemoglobin A1c (HbA1c) level of > 6.5% [20], or current use of hypoglycemic agents. Dyslipidemia was defined as current use of lipid-lowering agents, or a low-density lipoprotein cholesterol level of ≥ 140 mg/dl, triglyceride level of ≥ 150 mg/dl, or high-density lipoprotein cholesterol (HDL-cholesterol) level of < 40 mg/dl in a fasting blood sample. Hypertension was defined as a sitting blood pressure of ≥ 140/90 mmHg or current use of antihypertensive agents [21]. Smoking status was categorized as currently smoking or not smoking. BMI was defined as the body weight (kg) divided by the square of the height (m^2^). Venous blood samples were collected at the outpatient clinic after an overnight fast of 8–12 hours. Serum levels of lipids (total cholesterol, HDL-cholesterol, and triglycerides), HbA1c, and high-sensitivity C-reactive protein (hsCRP) were measured by routine methods using an AutoAnalyzer at the central laboratory of Okayama University Hospital.

### Statistical analysis

Continuous variables are presented as the mean ± standard deviation or median (interquartile range). Data that were not normally distributed according to the Kolmogorov–Smirnov test were logarithmically transformed before analysis. Categorical variables are presented as frequency (percentage). Continuous and categorical variables were compared between groups using the unpaired Student’s *t*-test or the chi-square test, as appropriate. Correlations between the VAT area and the numbers of areas with various plaque characteristics were determined using the Spearman rank correlation test. The VAT area was categorized into tertiles (T1, T2, and T3). For all patients, T1 ≤ 65.6 cm^2^ (n = 152), 65.6 cm^2^ < T2 ≤ 114.5 cm^2^ (n = 152), and T3 > 114.5 cm^2^ (n = 152). For patients with DM, T1 ≤ 87 cm^2^ (n = 40), 87 < T2 ≤ 130.0 cm^2^ (n = 41), and T3 > 130.0 cm^2^ (n = 41). For patients without DM, T1 ≤ 60 cm^2^ (n = 111), 60 < T2 ≤ 106 cm^2^ (n = 112), and T3 > 106 cm^2^ (n = 111). Multivariate logistic regression analyses were performed to assess whether the associations between vulnerable plaque and VAT area were independent of age, sex, hypertension, dyslipidemia, and current smoking. A p value < 0.05 was considered statistically significant. All statistical analyses were performed using SPSS 17.0 for Windows (SPSS Inc., Chicago, IL). Receiver operating characteristic (ROC) curve analysis was used to determine the specificity and sensitivity of the VAT area for differentiating between patients with and without vulnerable plaque.

## Results

Table 1 shows the clinical characteristics of subjects with and without DM. A total of 456 patients (57% male) with a mean age of 64 ± 14 years (range 16–92 years) were included in our analyses. Vulnerable plaques were detected in 17 (16%) of the 456 patients. Patients with DM were more likely to be male and had a higher BMI, larger WC, larger VAT area, higher prevalence of hypertension and dyslipidemia, and higher triglyceride level than patients without DM.

**Table 1:** Baseline characteristics of subjects with and without DM

	**All (n = 456)**	**Without DM (n = 334)**	**With DM (n = 122)**	**p**
Age (years)	64 ± 14	64 ± 15	66 ± 12	0.15
Men, n (%)	261 (57)	182 (54)	79 (65)	0.04
Body mass index (kg/m^2^)	23 ± 4	23 ± 3	25 ± 4	<0.01
Waist circumference (cm)	84 ± 11	82 ± 10	88 ± 11	<0.01
Subcutaneous adipose tissue (cm^2^)	136 ± 79	132 ± 76	147 ± 84	0.08
Visceral adipose tissue (cm^2^)	95 ± 55	88 ± 52	116 ± 58	<0.01
Hypertension, n (%)	263 (58)	176 (53)	87 (71)	<0.01
Dyslipidemia, n (%)	208 (46)	130 (39)	78 (66)	<0.01
Current smoking, n (%)	98 (21)	72 (22)	26 (21)	0.96
Total cholesterol (mg/dl)	187 ± 37	188 ± 38	187 ± 36	0.95
Triglycerides (mg/dl)	143 (88)	138 (107)	154 (121)	0.02
HDL-cholesterol (mg/dl)	57 ± 16	58 ± 16	56 ± 17	0.16
LDL-cholesterol (mg/dl)	111 ± 32	111 ± 33	110 ± 30	0.93
Hemoglobin A1c (%)	6.2 ± 1.2	5.7 ± 0.4	7.4 ± 1.6	<0.01
High sensitivity CRP (mg/dl)	0.16 (0.08)	0.15 (0.08)	0.17 (0.09)	0.30
Medications				
Antihypertensive agents, n (%)	239 (52)	160 (48)	79 (65)	<0.01
Lipid lowering agents n (%)	138 (30)	84 (25)	54 (44)	<0.01
Hypoglycemic agents, n (%)	82 (18)	0 (0)	82 (67)	<0.01

DM, diabetes mellitus; HDL, high-density lipoprotein; LDL, low-density lipoprotein; CRP, C-reactive protein.

Table 2 shows the baseline characteristics according to the VAT area tertile in patients with and without DM. In patients without DM, the T3 group had a higher proportion of men, more advanced age, higher prevalence of hypertension and use of antihypertensive agents, higher prevalence of dyslipidemia and use of lipid-lowering agents, lower level of HDL-cholesterol, and higher levels of triglycerides, HbA1c, and hsCRP than the T1 group. In patients with DM, the T3 group had a higher proportion of men, higher prevalence of hypertension and use of antihypertensive agents, and higher triglyceride level than the T1 group.

**Table 2:** Baseline characteristics according to the VAT area tertile in patients with and without DM

	**Without DM (n = 334)**			**With DM (n = 122)**		
**Characteristic**	**VAT T1 (n = 111)**	**VAT T2 (n = 112)**	**VAT T3 (n = 111)**	**VAT T1 (n = 40)**	**VAT T2 (n = 41)**	**VAT T3 (n = 41)**
Age (years)	58 ± 19	66 ± 13*	67 ± 11*	65 ± 14	67 ± 11	65 ± 12
Men, n (%)	45 (41)	60 (54)	77 (69)*†	23 (58)	21 (51)	35 (85)*†
Body mass index (kg/m^2^)	20 ± 2	23 ± 2*	25 ± 3*†	22 ± 3	25 ± 4*	27 ± 4*†
Waist circumference (cm)	74 ± 7	82 ± 6*	91 ± 8*†	80 ± 8	88 ± 9*	96 ± 10*†
Subcutaneous adipose tissue (cm^2^)	91 ± 63	131 ± 58*	175 ± 82*†	112 ± 72	151 ± 79*	177 ± 90*
Visceral adipose tissue (cm^2^)	35 ± 15	82 ± 13*	147 ± 39*†	55 ± 22	111 ± 13*	180 ± 41*†
Hypertension, n (%)	47 (42)	59 (53)	70 (63)*	23 (58)	31 (76)	33 (80)*
Dyslipidemia, n (%)	26 (23)	48 (43)*	56 (50)*	25 (63)	25 (61)	28 (68)
Current smoking, n (%)	18 (16)	25 (22)	29 (26)	11 (28)	6 (15)	9 (22)
Total cholesterol (mg/dl)	184 ± 39	192 ± 37	189 ± 38	185 ± 40	187 ± 34	189 ± 34
Triglycerides (mg/dl)	92 (51)	149 (88)*	169 (103)*	106 (46)	154 (78)	200 (165)*†
HDL-cholesterol (mg/dl)	63 ± 17	57 ± 15*	53 ± 15*	60 ± 19	54 ± 14	53 ± 19*
LDL-cholesterol (mg/dl)	108 ± 33	115 ± 32	109 ± 34	107 ± 28	113 ± 29	109 ± 34
Hemoglobin A1c (%)	5.5 ± 0.3	5.7 ± 0.4*	5.8 ± 0.5*†	7.4 ± 1.6	7.5 ± 1.6	7.4 ± 1.7
High sensitivity CRP (mg/dl)	0.16 (0.14)	0.15 (0.12)	0.16 (0.13)*	0.14 (0.11)	0.16 (0.20)	0.20 (0.22)
Medications						
Antihypertensive agents, n (%)	44 (40)	50 (45)	66 (59)*†	21 (53)	27 (66)	31 (76)*
Lipid-lowering agents, n (%)	15 (14)	32 (29)*	37 (33)*	20 (50)	11 (27)*	23 (56)†
Hypoglycemic agents, n (%)	0 (0)	0 (0)	0 (0)	22 (55)	29 (71)	30 (73)

DM, diabetes mellitus; HDL, high-density lipoprotein; LDL, low-density lipoprotein; HbA1c, hemoglobin A1c.

The VAT area data were divided into tertiles (T) as described in the Methods. *p < 0.05 vs. T1 group, †p < 0.05 vs. T2 group.

**Figure 1:**
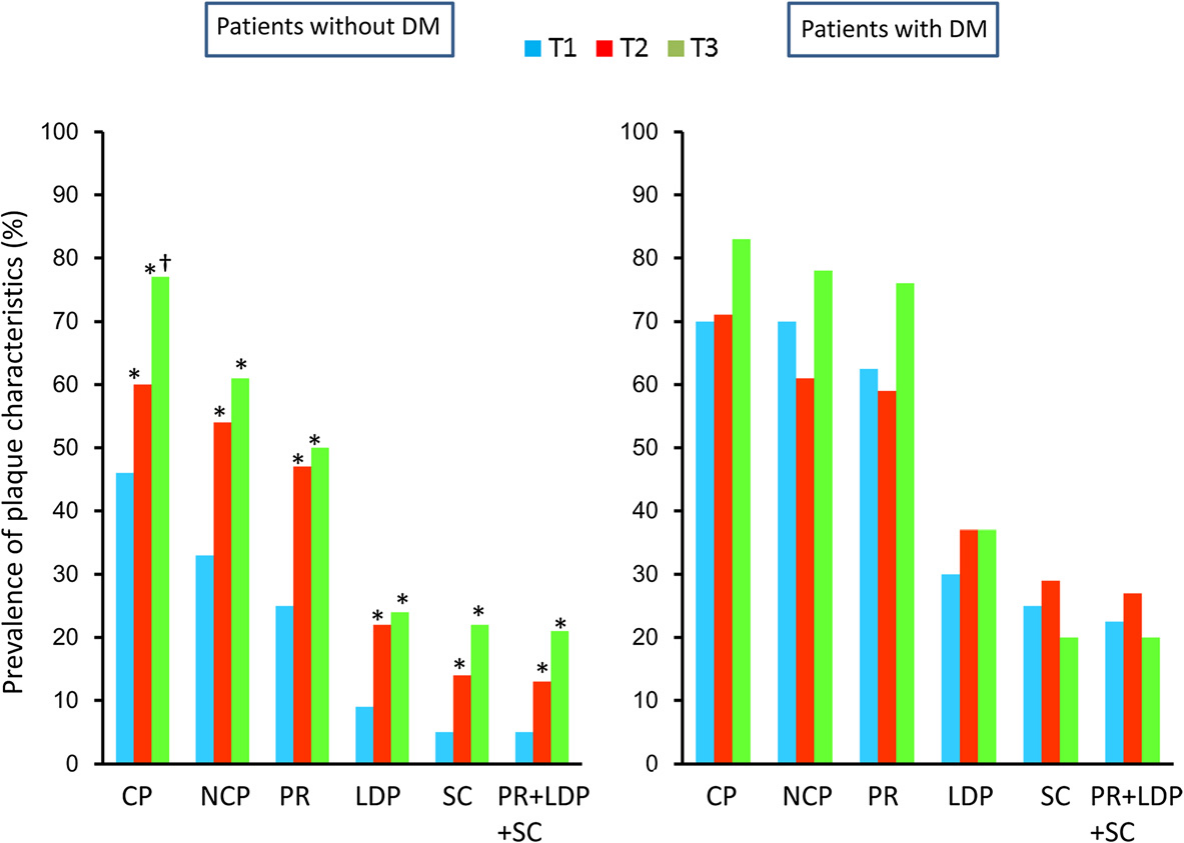
**Prevalences of plaque characteristics according to VAT area tertile in patients with and without DM.** CP, calcified plaque; NCP, noncalcified plaque; PR, positive remodeling; LDP, low-density plaque; SC, spotty calcification. *p < 0.05 vs. T1 group; †p < 0.05 vs*.* T2 group.

**Figure 2:**
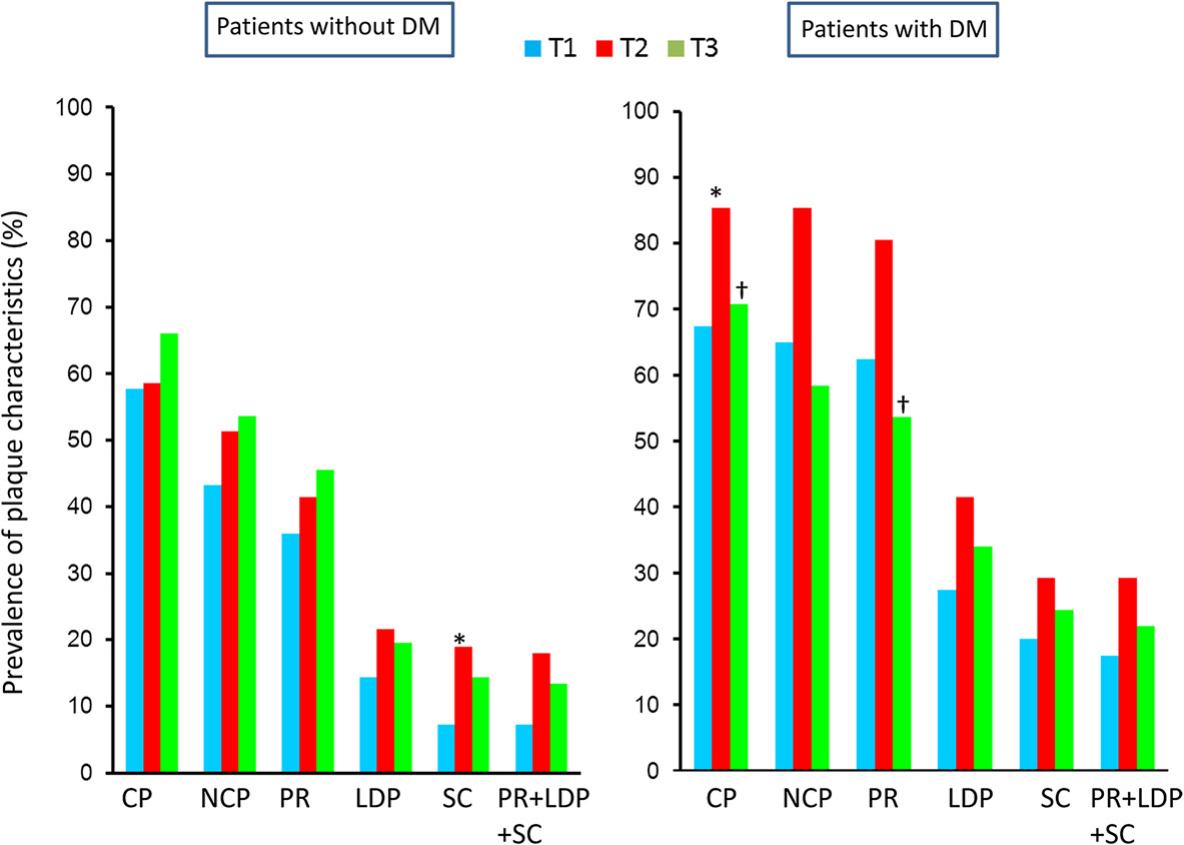
**Prevalences of plaque characteristics according to BMI tertile in patients with and without DM.** CP, calcified plaque; NCP, noncalcified plaque; PR, positive remodeling; LDP, low-density plaque; SC, spotty calcification. *p < 0.05 vs. T1 group; †p < 0.05 vs*.* T2 group.

**Figure 3:**
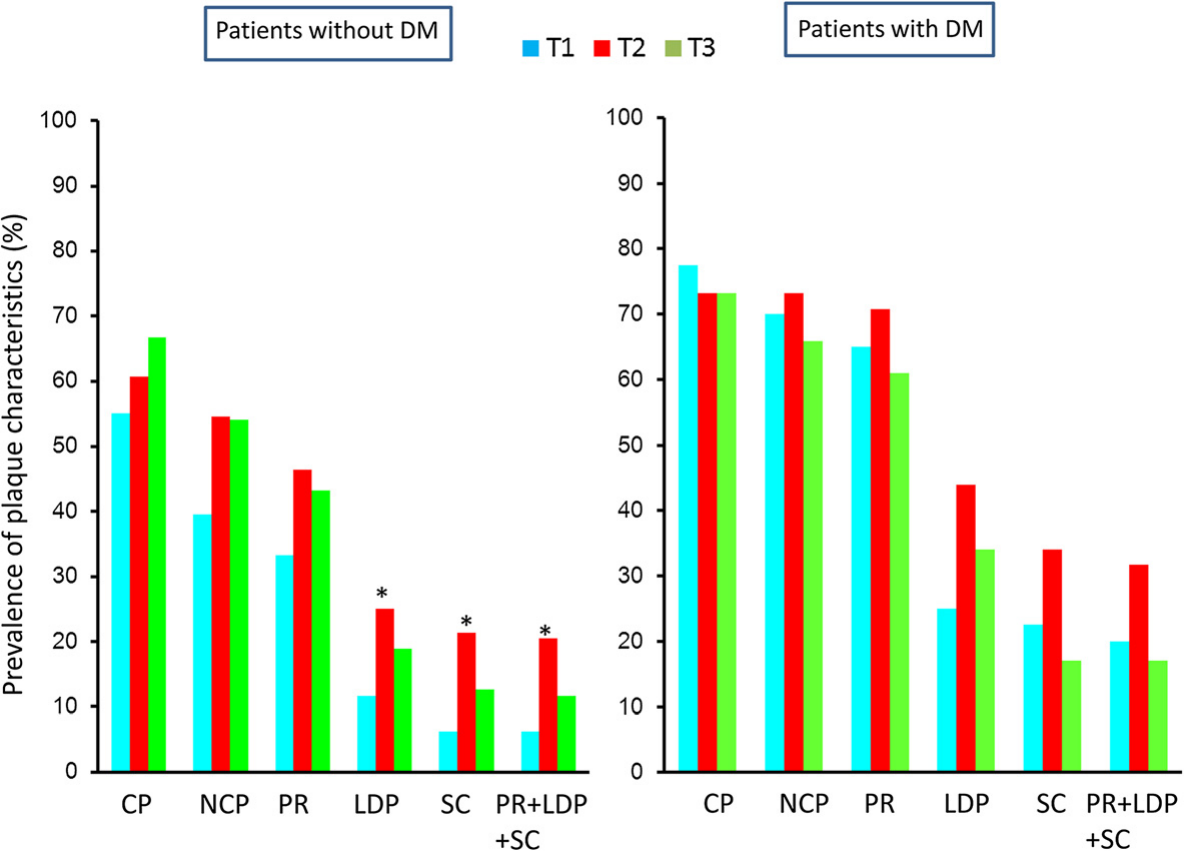
**Prevalences of plaque characteristics according to WC tertile in patients with and without DM.** CP, calcified plaque; NCP, noncalcified plaque; PR, positive remodeling; LDP, low-density plaque; SC, spotty calcification. *p < 0.05 vs. T1 group.

**Figure 4:**
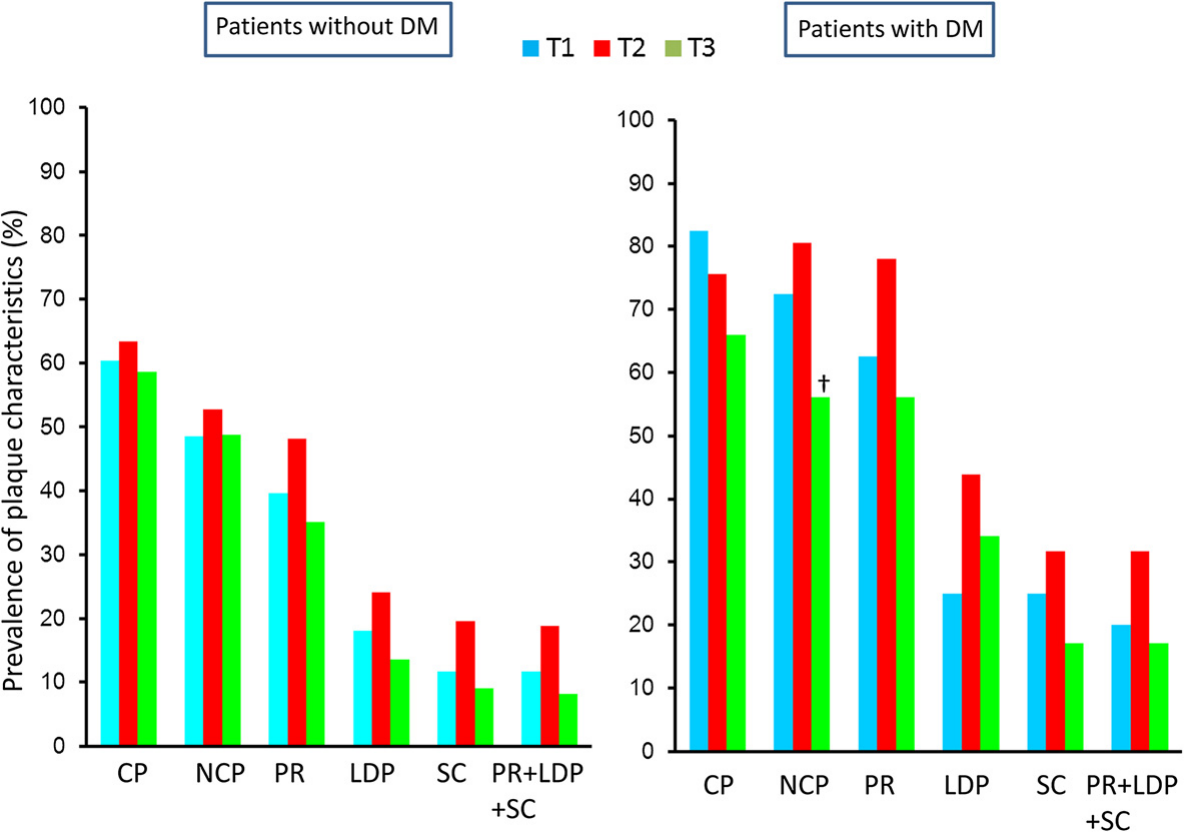
**Prevalences of plaque characteristics according to SAT tertile in patients with and without DM.** CP, calcified plaque; NCP, noncalcified plaque; PR, positive remodeling; LDP, low-density plaque; SC, spotty calcification. †p < 0.05 vs*.* T2 group.

Figure 1 shows the prevalence of plaque characteristics according to the VAT area tertile in patients with and without DM. In patients with DM, there were no significant differences in the prevalence of any of the plaque characteristics among the tertiles. In patients without DM, there was a stepwise increase in the prevalence of each plaque characteristics from the T1 group to the T3 group. The T1 group had a lower prevalence than the T2 and T3 groups of positive remodeling (25 vs. 47 and 50%, p < 0.01), low-density plaques (9 vs. 22 and 24%, p < 0.01), adjacent areas of spotty calcification (5 vs. 14 and 22%, p = 0.01), and all three of these characteristics (5 vs. 14 and 21%, p = 0.02). Figure 2 shows the prevalence of plaque characteristics according to the BMI tertile in patients with and without DM. In patients without DM, there were no significant differences in the prevalence of calcified plaque, NCP, or positive remodeling among the tertiles. In patients with DM, the T2 group tended to have a higher prevalence of calcified plaque and positive remodeling than the T1 and the T3 groups. There was no significant difference in the prevalence of vulnerable plaques among the BMI tertiles. Figure 3 shows the prevalence of plaque characteristics according to the WC tertile in patients with and without DM. In patients without DM, the T1 group had a significantly lower prevalence of vulnerable plaque than the T2 group. Patients with DM had a similar trend in terms of the prevalence of vulnerable plaque among tertiles, but the differences were not significant. Figure 4 shows the prevalence of plaque characteristics according to the SAT tertile in patients with and without DM. In patients with and without DM, there was no significant difference in the prevalence of vulnerable plaque among tertiles.

Table 3 shows the numbers of plaques with various characteristics according to the VAT area tertile in patients with and without DM. The numbers of calcified plaques, NCPs, mixed plaques, vulnerable plaques, and significant stenoses were significantly higher in the T3 group than in the T1 group in patients without DM. There was a stepwise increase in the coronary artery calcium score from the T1 group to the T3 group in patients without DM, but not in patients with DM. This association remained significant after adjustment for age (Table 4).

**Table 3:** Numbers of plaques with various characteristics according to VAT area tertiles in patients with and without DM

	**Without DM (n = 334)**			**With DM (n = 122)**		
**Characteristics**	**VAT T1 (n = 111)**	**VAT T2 (n = 112)**	**VAT T3 (n = 111)**	**VAT T1 (n = 40)**	**VAT T2 (n = 41)**	**VAT T3 (n = 41)**
Calcified plaque	1.7 ± 2.3	2.1 ± 2.7	3.0 ± 3.1*†	3.4 ± 3.4	3.3 ± 3.3	4.8 ± 3.7
Noncalcified plaque	0.7 ± 1.3	1.3 ± 1.6*	1.6 ± 2.0*	2.1 ± 2.2	2.0 ± 2.2	2.4 ± 2.1
Mixed plaque	0.5 ± 0.9	0.9 ± 1.2*	1.2 ± 1.7*	1.3 ± 1.6	1.3 ± 1.9	1.7 ± 1.8
Vulnerable plaque	0.2 ± 0.6	0.3 ± 0.5	0.3 ± 0.7*	0.6 ± 1.1	0.6 ± 1.0	0.5 ± 0.8
Significant stenosis	0.3 ± 0.7	0.3 ± 0.8	0.6 ± 1.3*†	0.6 ± 1.1	0.6 ± 1.2	0.8 ± 1.2
Coronary artery calcium score	177 (156)	207 (145)	307 (270)	461 (746)	380 (241)	495 (958)

*p < 0.05 vs. T1 group; †p < 0.05 vs. T2 group.

**Table 4:** Age-adjusted associations between numbers of plaques with various characteristics and VAT area

**Dependent variable: visceral adipose tissue area**	**Without DM (n = 334)**		**With DM (n = 122)**	
	**ρ**	**p**	**ρ**	**p**
Calcified plaque	0.12	0.03	0.08	0.37
Noncalcified plaque	0.16	<0.01	-0.01	0.89
Mixed plaque	0.15	<0.01	0.05	0.61
Vulnerable plaque	0.15	<0.01	-0.03	0.78

Table 5 shows associations between vulnerable plaque and clinical variables and VAT area tertiles in patients with and without DM. In patients without DM, univariate analysis showed that VAT area T2 and T3, older age, male sex, current smoking, and lower HDL-cholesterol level were significantly associated with vulnerable plaque. Multivariate analysis showed that VAT area T3 was significantly associated with vulnerable plaque even after adjustment for confounding factors in patients without DM (odds ratio 3.17, 95% confidence interval [CI] 1.08–9.31, p = 0.04; Table 6). In patients with DM, VAT tertiles were not associated with vulnerable plaque (Table 6).

**Table 5:** Factors associated with vulnerable plaque in patients with and without DM

	**Without DM (n = 334)**		**With DM (n = 122)**	
**Factor**	**Odds ratio (95% CI)**	**p**	**Odds ratio (95% CI)**	**p**
Age (per year)	1.04 (1.01–1.07)	<0.01	1.02 (0.99–1.07)	0.22
Male sex	5.11 (2.20–11.85)	<0.01	1.86 (0.72–4.82)	0.20
Hypertension	1.61 (0.83–3.11)	0.16	1.64 (0.60–4.46)	0.34
Dyslipidemia	1.42 (0.74–2.71)	0.28	1.25 (0.51–3.07)	0.62
Current smoking	3.16 (1.61–6.20)	<0.01	0.76 (0.26–2.23)	0.61
Total cholesterol^a^	0.80 (0.02–30.28)	0.90	0.10 (0.001–21.32)	0.40
Triglycerides^a^	2.39 (0.64–8.87)	0.19	2.48 (0.39–15.87)	0.34
HDL-cholesterol^a^	0.025 (0.001–0.49)	0.02	0.43 (0.016–11.25)	0.61
LDL-cholesterol^a^	6.50 (0.44–96.74)	0.17	0.13 (0.003–4.84)	0.27
Hemoglobin A1c	1.86 (0.88–3.91)	0.10	1.02 (0.78–1.33)	0.90
High sensitivity CRP^a^	1.04 (0.50-2.18)	0.91	1.36 (0.52-3.55)	0.53
VAT T1	1.00	1.00	1.00	1.00
VAT T2	3.28 (1.15–9.36)	0.03	1.26 (0.46–3.48)	0.65
VAT T3	5.54 (2.02–15.18)	<0.01	0.84 (0.29–2.44)	0.74
Antihypertensive agents	1.29 (0.68–2.46)	0.43	0.97 (0.40–2.35)	0.95
Lipid-lowering agents	1.34 (0.66–2.71)	0.41	1.63 (0.70–3.80)	0.26
Hypoglycemic agents	Not available		1.09 (0.44–2.68)	0.85

The VAT area data were divided into tertiles (T) as described in the Methods.

^a^Values were log-transformed before analysis.

DM, diabetes mellitus; CI, confidence interval; HDL, high-density lipoprotein; LDL, low-density lipoprotein; VAT, visceral adipose tissue.

**Table 6:** Associations between adiposity measurements and vulnerable plaque

	**Without DM (n = 334)**				**With DM (n = 122)**	
	**Univariate**		**Multivariate**		**Univariate**	
	**Odds ratio (95% CI)**	**p**	**Odds ratio (95% CI)**	**p**	**Odds ratio (95% CI)**	**p**
Visceral adipose tissue:						
T1	1				1	
T2	3.28 (1.15–9.36)	0.03	2.32 (0.77–6.97)	0.13	1.26 (0.46–3.48)	0.65
T3	5.54 (2.02–15.18)	<0.01	3.17 (1.08–9.31)	0.04	0.84 (0.29–2.44)	0.74
Subcutaneous adipose tissue:						
T1	1				1	
T2	1.74 (0.82–3.68)	0.14	1.81 (0.81–4.05)	0.15	1.86 (0.67–5.13)	0.23
T3	0.67 (0.27–1.63)	0.37	1.14 (0.43–3.04)	0.8	0.82 (0.27–2.53)	0.73
Body mass index:						
T1	1				1	
T2	2.83 (1.19–6.74)	0.02	3.34 (1.30–8.58)	0.01	1.95 (0.68–5.62)	0.22
T3	1.99 (0.81–4.91)	0.13	1.99 (0.75–5.26)	0.17	1.33 (0.44–3.99)	0.62
Waist circumference:						
T1	1				1	
T2	3.84 (1.57–9.37)	<0.01	3.43 (1.34–8.77)	0.01	1.86 (0.67–5.13)	0.23
T3	1.97 (0.76–5.15)	0.17	1.77 (0.64–4.90)	0.27	0.82 (0.27–2.53)	0.73

The visceral adipose tissue area was divided into tertiles (T) as described in the Methods. The subcutaneous adipose tissue area was divided into tertiles as follows. In patients with DM, T1 ≤ 110 cm^2^ (n = 40), 110 < T2 ≤ 155 cm^2^ (n = 41), T3 > 155 cm^2^ (n = 41). In patients without DM, T1 ≤ 92 cm^2^ (n = 111), 92 < T2 ≤ 150 cm^2^ (n = 112), T3 > 150 cm^2^ (n = 111). The body mass index was divided into tertiles as follows. In patients with DM, T1 ≤ 22.72 kg/m^2^ (n = 40), 22.72 < T2 ≤ 25.2 kg/m^2^ (n = 41), T3 > 25.2 kg/m^2^ (n = 41). In patients without DM, T1 ≤ 21.15 kg/m^2^ (n = 111), 21.15 < T2 ≤ 24.06 kg/m^2^ (n = 111), T3 > 24.06 kg/m^2^ (n = 112). The waist circumference was divided into tertiles as follows. In patients with DM, T1 ≤ 81.5 cm (n = 40), 81.5 cm < T2 ≤ 91 cm (n = 41), T3 > 91 cm (n = 41). In patients without DM, T1 ≤ 77 cm (n = 111), 77 < T2 ≤ 86 cm (n = 112), T3 > 86 cm (n = 111). Multivariate analyses were adjusted for age, male sex, hypertension, dyslipidemia, and current smoking.

DM, diabetes mellitus; CI, confidence interval.

Table 6 shows the associations between adiposity variables and vulnerable plaque. In patients without DM, VAT area T3, BMI T2, and WC T2 were independently associated with vulnerable plaque after adjustment for age, sex, smoking, hypertension, and dyslipidemia. There were no associations between adiposity and vulnerable plaque in patients with DM.

ROC curve analysis of the VAT area in patients without DM showed an optimal cutoff value of 91.3 cm^2^ for identification of patients with vulnerable plaque, with sensitivity of 70%, specificity of 62%, and area under the curve of 0.69 (95% CI 0.61–0.77, p < 0.01). In patients with DM, the area under the curve was 0.48 (95% CI 0.37–0.58, p = 0.71) (data not shown).

## Discussion

The results of this study show that a large VAT area is associated with various characteristics of vulnerable coronary artery plaques on CTA in patients without DM, but not in patients with DM. The prevalence of vulnerable plaques was similar in all VAT area tertiles of patients with DM (about 20%) and in the highest VAT area tertile in patients without DM. These findings support the hypothesis that a large VAT area is a cardiometabolic risk factor that is significantly associated with vulnerable plaque before the development of DM, and that measurement of the VAT area is useful for assessing cardiovascular risk in patients without DM.

The results of this study show a positive association between a large VAT area and the NCP burden on CTA in patients without DM, but not in patients with DM. Several previous studies reported that the VAT area was an independent marker of CAD [13,22]. Excess VAT triggers insulin resistance, which may be accompanied by release of inflammatory mediators and cytokines from dysfunctional adipose tissue and may be strongly associated with the formation and progression of coronary artery plaques. The different associations of the VAT area with characteristics of coronary plaques in different groups in this study may be explained by variations in HDL-cholesterol and HbA1c levels, which are known to be associated with cardiovascular risk [23,24]. In patients without DM, the HDL- cholesterol and HbA1c levels changed unfavorably with increasing VAT area tertiles, whereas in patients with DM, these factors were not significantly different among the VAT area tertiles. The hsCRP level was significantly higher in the VAT area T3 group than in the T1 and T2 groups in patients without DM, but was not significantly different among tertiles in patients with DM. Another explanation is that hyperglycemia may be the main factor determining cardiovascular risk in patients with DM, and may override the effects of excess VAT-dependent inflammatory mediators and cytokines. Oxidative stress caused by hyperglycemia [25] and advanced glycation end products [26] may have greater effects on plaque characteristics in patients with DM than in patients without DM, and may attenuate the impact of the VAT area. However, our findings are not consistent with those of a cross-sectional study which reported that the amount of VAT was strongly associated with cardiometabolic risk factors regardless of type 2 diabetes status [27]. This difference may be partly owed to differences in ethnicity of the study populations. The previous study was conducted in a population that included only 8.2% East Asian individuals, and did not include patients with suspected CAD, both of which were characteristics of our study subjects. Another study reported that the amount of VAT was not an independent coronary risk factor after adjustment for multiple covariates, although VAT was one of the variables influencing the development of atherosclerosis [28]. This is consistent with our hypothesis that visceral obesity is an important coronary risk factor, but precedes the development of DM. In patients without DM in this study, hypertension, dyslipidemia, and high triglyceride level tended to be associated with vulnerable plaque, but these associations were not statistically significant. A large amount of VAT increased the risks of cardiovascular risk factors such as hypertension and dyslipidemia, but these factors may not have sufficient individual impact to lead to the development of vulnerable coronary plaques. In other words, the amount of VAT is considered to be a representative marker of cardiometabolic risk, and may therefore be associated with plaque characteristics.

In this study, the highest tertile of the VAT area included a higher proportion of men than the other tertiles in patients with and without DM. Although there were no significant differences between males and females in the majority of cardiovascular risk factors, HDL and triglyceride levels have been reported to have a greater impact on coronary artery disease in women than in men [29]. In addition, smoking and inflammation (detected by a high CRP level) have been reported to have a more negative influence on coronary artery disease in women than in men. These sex differences may affect the prevalence of vulnerable plaque in different tertiles in patients without DM. However, our finding that there was a similar proportion of men in the highest tertile of the VAT area in patients with and without DM suggests that sex differences did not have a great impact on the associations between VAT and plaque characteristics.

The results of this study show associations between vulnerable coronary artery plaques and adiposity measurements on CT. VAT area T3, BMI T2, and WC T2 were significantly associated with vulnerable plaque in patients without DM. A previous study reported that a large VAT area was significantly associated with both the presence and extent of NCPs, whereas BMI and WC were not [13]. One possible reason for this difference is that they did not analyze these factors separately in patients with and without DM.

It is easier to evaluate adipose tissue attenuation and volume on CT than on ultrasonography [30]. Recently, the quality of abdominal fat attenuation has been reported to be associated with cardiometabolic risk, suggesting that evaluation of adipose tissue quality as well as quantity may be useful [31]. Other studies reported that epicardial adipose tissue was independently associated with coronary artery disease and coronary plaque characteristics [32-35]. Further studies are needed to clarify the associations between deposition of various adipose tissues and coronary plaque characteristics.

Several limitations of this study should be considered. First, this study was a single-center, retrospective study that included only 456 Japanese patients with suspected CAD. These subjects had a higher prevalence of risk factors than the general population, and the results may not be applicable to the general population or to other ethnicities. Further investigation in a larger population is needed to definitively determine the associations among VAT, vulnerable plaque, and DM. Second, adipocytokine levels were not measured, and this study was unable to determine a causal relationship between the amount of VAT and plaque vulnerability. However, these biomarkers are under investigation, and the lack of adipocytokine data does not affect the relationship between VAT and vulnerable plaque shown by this study. Third, we excluded all patients with a history of coronary artery stenting or coronary artery bypass graft surgery because of the unreliability of coronary plaque assessment by CTA in such patients. Although this resulted in exclusion of 9% of the patients from our original study population, this probably had minimal effects on the analyses. As the accuracy of detection of obstructive CAD is decreased in patients with a high coronary artery calcium score [36,37], there is concern that the prevalence of vulnerable plaque is underestimated in the higher VAT tertiles of patients with DM. However, our finding that there were no significant differences in coronary artery calcium scores among the different tertiles in patients with DM may alleviate concerns over this issue. Finally, the latest consensus document on coronary CTA [38] states that plaque characteristics in coronary CTA are not well established. Even though this study assessed plaque characteristics as previously reported [18], substantial additional technical developments will be required to define the usefulness of these characteristics in terms of patient management.

In conclusion, the results of this study show that a large VAT area is associated with characteristics of vulnerable coronary plaques on CTA in patients without DM, but not in patients with DM. Our findings support the hypothesis that VAT is a significant cardiometabolic risk factor that is associated with vulnerable plaque before the development of DM. CTA findings may help to improve risk stratification in such patients.

## Abbreviations

BMI: Body mass index; CAD: Coronary artery disease; CI: Confidence interval; CTA: Computed tomography angiography; DM: Diabetes mellitus; HbA1c: Hemoglobin A1c; HDL- cholesterol: High-density lipoprotein cholesterol; LDL-cholesterol: Low-density lipoprotein cholesterol; NCP: Noncalcified plaque; ROC: Receiver operating characteristic; SAT: Subcutaneous adipose tissue; VAT: Visceral adipose tissue; WC: Waist circumference.

## Competing interests

The authors declare that they have no competing interests.

## Authors’ contributions

KO collected and analyzed the data and wrote the manuscript. TM conceived and designed the study. YK, NA, YM, MK, and HK contributed to collection and analysis of the data. SS, KN, HM, SK, and HI contributed to the study design and discussion. All authors read and approved the final manuscript.
